# Atomic resolution snapshot of *Leishmania* ribosome inhibition by the aminoglycoside paromomycin

**DOI:** 10.1038/s41467-017-01664-4

**Published:** 2017-11-17

**Authors:** Moran Shalev-Benami, Yan Zhang, Haim Rozenberg, Yuko Nobe, Masato Taoka, Donna Matzov, Ella Zimmerman, Anat Bashan, Toshiaki Isobe, Charles L. Jaffe, Ada Yonath, Georgios Skiniotis

**Affiliations:** 1 Faculty of Chemistry, Department of Structural Biology, Weizmann Institute of Science, Rehovot, 761001 Israel; 2 Departments of Molecular and Cellular Physiology, and Structural Biology, Stanford University School of Medicine, Stanford, CA 94305 USA; 3 Graduate School of Science and Engineering, Tokyo Metropolitan University, Hachioji-shi, Tokyo 192-0397 Japan; 4 Department of Microbiology and Molecular Genetics, IMRIC, Hebrew University-Hadassah Medical School, Jerusalem, 91120 Israel

## Abstract

*Leishmania* is a single-celled eukaryotic parasite afflicting millions of humans worldwide, with current therapies limited to a poor selection of drugs that mostly target elements in the parasite’s cell envelope. Here we determined the atomic resolution electron cryo-microscopy (cryo-EM) structure of the *Leishmania* ribosome in complex with paromomycin (PAR), a highly potent compound recently approved for treatment of the fatal visceral leishmaniasis (VL). The structure reveals the mechanism by which the drug induces its deleterious effects on the parasite. We further show that PAR interferes with several aspects of cytosolic translation, thus highlighting the cytosolic rather than the mitochondrial ribosome as the primary drug target. The results also highlight unique as well as conserved elements in the PAR-binding pocket that can serve as hotspots for the development of novel therapeutics.

## Abstract (web summary)

Leishmaniasis is a parasitic disease transmitted by the bite of infected sand flies. Here the authors describe an atomic resolution cryo-EM structure of the *Leishmania* ribosome in complex with the recently approved drug paromomycin (PAR) and highlight conserved elements in the drug binding pocket that mediate PAR deleterious effects on the parasite.

## Introduction

Leishmaniasis, a parasitic disease transmitted by the bite of infected sand flies, afflicts over 12 million people in nearly 100 endemic countries^1^. The disease is caused by more than 20 pathogenic species of eukaryotic microbes that parasitize macrophage populations residing in the skin, mucus or visceral organs. These infections result in a relatively large array of clinical manifestations defined as cutaneous-, mucosal-, or visceral leishmaniasis, respectively^1^. Currently, there is no vaccine against leishmaniasis and the therapeutic arsenal to treat the disease is restricted by both the limited drugs available and the emergence of parasite resistance mechanisms^1^.

Paromomycin (PAR), a natural aminoglycoside (AG) produced by *Streptomyces riomosus*, was recently approved for the treatment of visceral leishmaniasis (VL), a fatal form of leishmanial infection^2^. PAR is a highly potent antibacterial agent known to confer a broad spectrum of activities against both prokaryotic and eukaryotic microbes^3^. PAR mechanisms of action in bacteria are well documented and are mainly attributed to interfering with the bacterial translation apparatus^4^. By contrast, very little is known about PAR actions on eukaryotic microbes, where it has been proposed to interfere with mitochondrial translation^5^.

Here we employed a combination of structural and biochemical approaches to study PAR activities against the parasite *Leishmania donovani* (*L. donovani*), the main cause of VL^6^. Our biochemical results, showing a strong correlation between interference with cytosolic ribosome translation and inhibition of parasite growth, suggest the cytosolic rather than mitochondrial ribosome as the main drug target. Cryo-EM analysis of the fully active cytosolic *Leishmania* ribosome in complex with PAR enabled us to obtain the structure at atomic resolution, thereby providing a detailed snapshot of the drug’s binding pocket and the ribosome decoding center. The results reveal unique elements within the pocket that contribute to drug binding in eukaryotic microbes, and coupled to biochemical and in silico experiments highlight additional members of the AG family as potential inhibitors of leishmanial cytosolic translation.

## Results

### AGs interfere with the leishmanial cytosolic translation

Although AGs are extensively used as anti-parasitic agents, the identity of their target in eukaryotic microorganisms has been unclear. Based on the similarity of key elements shared between the AG-binding pocket in bacteria and the corresponding site in the mitochondrial ribosome, namely the presence of an adenine residue at position 1408 (Supplementary Fig. 1), the mitochondrial translation machinery has been considered to be the main target for these compounds in eukaryotic parasites^5^. However, recent structural studies demonstrating that AGs can bind to synthetic RNA constructs mimicking their putative binding site in cytosolic ribosomes have raised the possibility that the leishmanial cytosolic ribosome might also be the target of AGs in *Leishmania* ^7–9^. Given the lack of direct correlation between the inhibition of cytosolic or mitochondrial translation and parasite growth, the main target of AGs in the parasitic cell is yet to be determined.

**Fig. 1:**
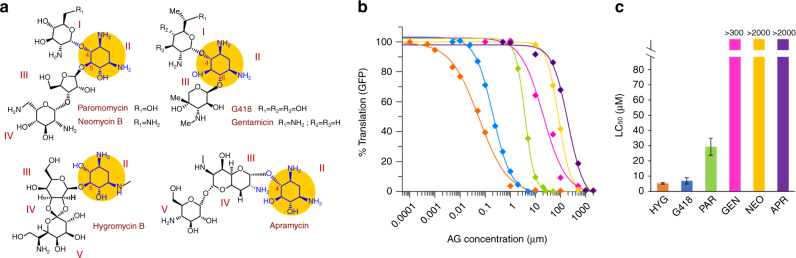
Aminoglycoside effects on *Leishmania* and the leishmanial translation machinery. **a** Chemical structures of AGs used in the study. Ring numbers are indicated in red; the common 2-deoxystreptamine ring (ring II) is highlighted in yellow and the substitution patterns in derivatized compounds are given as R_1_ and R_2_. **b** In vitro inhibition of cytosolic ribosome translation in *L. tarentolae* lysates. Each value represents the mean ± standard error of at least three independent experiments performed in duplicates. Hygromycin B (HYG, orange), Geneticin (G418, blue), Paromomycin (PAR, green), Gentamicin (GEN, pink), Neomycin (NEO, yellow), and Apramycin (APR, purple). **c** Inhibition of *L. donovani* promastigote growth by AGs. Values indicate drug concentrations inducing 50% inhibition of parasite growth (LC_50_). Each value represents the mean ± standard error of at least three independent experiments performed in triplicates

In order to assess whether AGs target the cytosolic ribosome in *Leishmania*, we measured the interference of six structurally diverged AGs (Fig. 1a) with leishmanial cytosolic ribosome translation and their effect on parasite growth. The selected derivatives were predicted to have differential selectivity for eukaryote cytosolic translation based on previous observations in higher eukaryotes^10^. The in vitro inhibition pattern obtained for the leishmanial cytosolic ribosome shows a diverse range of induced activities (Fig. 1b) with 4 orders of magnitude differentiating between the most potent compound, hygromycin B (HYG), and the least active derivative, apramycin (APR) (calculated IC_50_ values are 0.065, 0.15, 3.62, 18.4, 82.4, and 200.6 μM for HYG, G418, PAR, gentamicin (GEN), neomycin B (NEO) and APR, respectively; Table 1). The inhibition of parasite growth was strikingly correlated with the in vitro assay results (Fig. 1c), indicating the strong involvement of cytosolic ribosome inhibition by AGs with the induced anti-parasitic effects.

**Table 1:** In vitro inhibition of translation measured in *L. tarentolae* ^a^, *E. coli* ^b^, and rabbit^c^ reticulocyte lysates

Inhibition of translation—IC_50_ (μM)			
	*Leishmania* ^a^	Bacteria^b^	Reticulocytes^c^
G418	0.153 ± 0.0356	0.009 ± 0.0015^9^	2 ± 0.3^9^
GEN	18.41 ± 3.139	0.028 ± 0.004	62 ± 9
PAR	3.62 ± 0.176	0.051 ± 0.005^9^	57 ± 4^9^
NEO	82.36 ± 2.139	0.011 ± 0.001	28 ± 5
APR	200.6 ± 16.12	0.049 ± 0.003	29 ± 6
HYG	0.0647 ± 0.0053	0.011 ± 0.002	0.8 ± 0.1

Each value represents the mean ± SE of at least three independent repeats

G418, geneticin; GEN, gentamicin; PAR, paromomycin; NEO, neomycin; APR, apramycin; HYG, hygromycin B

### PAR binds the decoding center of the leishmanial ribosome

**Fig. 2:**
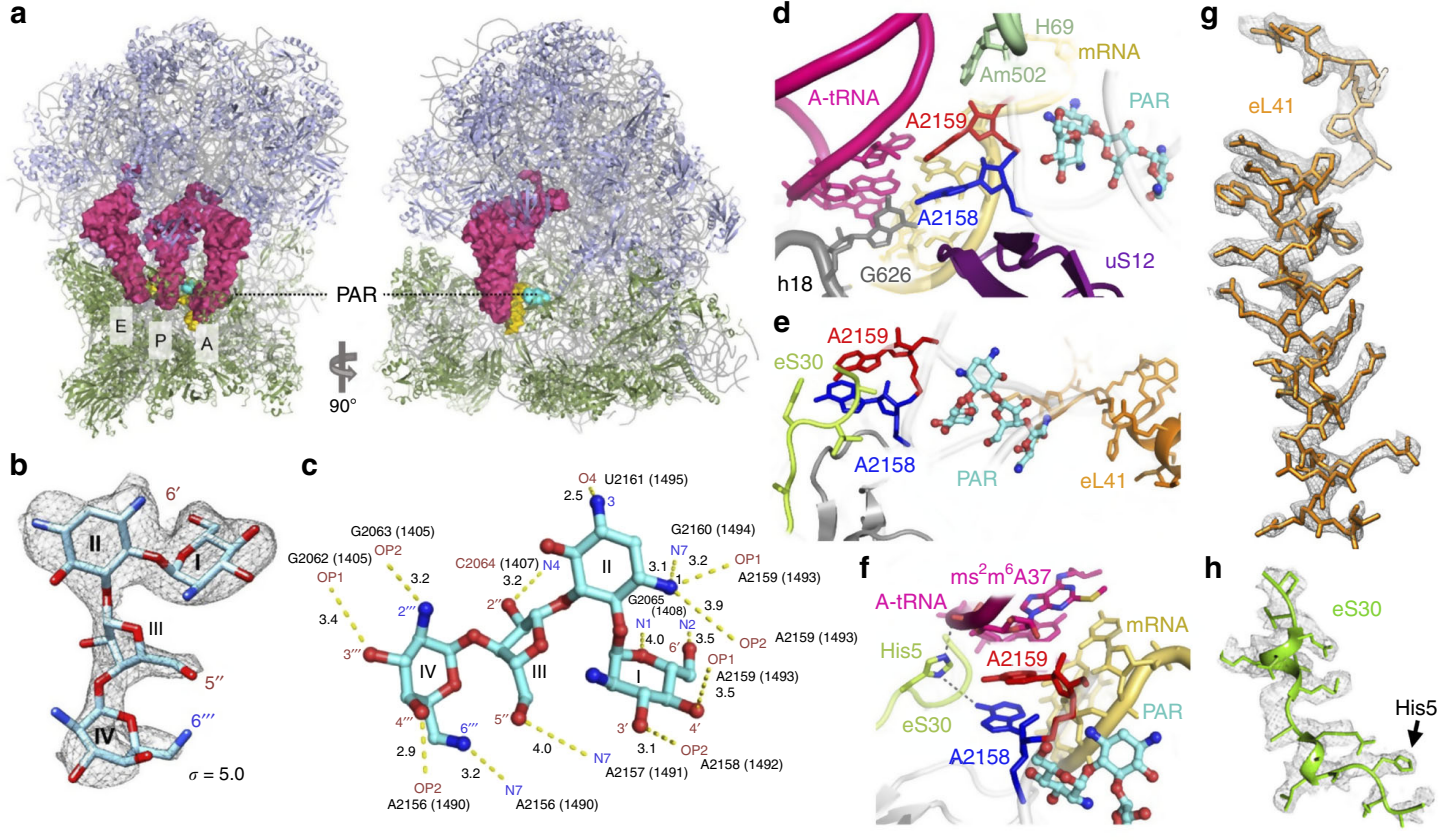
AGs target the decoding center of the leishmanial cytosolic ribosome. **a** An overall view of the cryo-EM structure with three tRNAs at their designated locations at the A-, P-, and E-sites (pink) and mRNA (yellow). PAR is shown in cyan, rRNA in gray, and ribosomal proteins in green and light blue for the small and large subunits, respectively. **b** Close-up view of PAR in density map. Observed density is contoured around the ligand at 5.0 *σ*. **c** PAR interactome within its binding pocket at the leishmanial ribosome. PAR maintains a dense array of electrostatic interactions with RNA residues within the binding pocket at the SSU. Ring numbers (I–IV) for PAR and atom names are specified. rRNA atoms are numbered according to the *Leishmania* numbering (with *E. coli* numbering in parenthesis). Hydrogen bonds and salt bridges are presented as yellow dashed lines. Bond lengths are presented in black in ångström (Å). **d** The PAR-binding pocket in the leishmanial cytosolic ribosome corresponds to the AG target in bacterial ribosomes and is localized at the tip of h44 at the SSU decoding center. Similar to bacteria, PAR binding dictates the flipped-out conformation of two highly conserved adenine residues, A2158 and A2159, blue and red, respectively (A1492 and A1493 in *E*. *coli* numbering), resulting in interactions with both the mRNA A-site codon (yellow) and the A-site tRNA anticodon loop (magenta). Additional conserved elements within the binding pocket include the universally conserved protein uS12 (purple) G626 of h18 (G530 in *E. coli* numbering, gray) and Am502β of LSU H69 (A1913 in *E. coli*, light green). **e** The ribosomal proteins eS30 (lime) and eL41 (orange), which are of sole eukaryotic origin, penetrate the drug binding pocket and maintain close interactions with binding site surroundings. **f** The eukaryote conserved His5 of eS30 (lime) maintains electrostatic interactions with both universally conserved A2158 (blue) and ms^2^m^6^A37 of A-site tRNA anticodon loop (magenta). **g**, **h** Eukaryotic proteins in the binding pocket that could not be resolved in earlier published structures due to regional flexibility are shown in density. eL41 (**g**) highlighted in orange, and eS30 (**h**) in green. Densities are shown at 3.0 *σ*

To delineate the PAR mechanism of action in *Leishmania* we employed cryo-EM to obtain the structure of intact cytosolic ribosomes derived from *L. donovani* in complex with the compound (Fig. 2). The nominal map resolutions for SSU and LSU were 2.7 and 2.5 Å, respectively (Supplementary Fig. 2). Importantly, the resolution of the map extended to 2.2 Å in the core regions, enabling us to build and refine an atomic model for the entire assembly including programed mRNA that was anchored to the ribosome via the leishmanial kozak sequence^11^ as well as three tRNA molecules bound at their designated A-, P-, and E- positions (Fig. 2, Supplementary Tables 1 and 2). A well-defined density that could accommodate the PAR four-ringed structure was found at the tip of SSU h44 at the ribosome decoding center (Fig. 2) in a binding pocket that corresponds to the AG primary binding site in bacterial ribosomes (Fig. 2, Supplementary Fig. 3). The high resolution of the map enabled the complete characterization of all four PAR sugar rings including the directionality of ring substituents, thus facilitating a full description of PAR interaction pattern within the *Leishmania* binding pocket (Fig. 2c, Supplementary Fig. 3). The structure reveals shared as well as distinct structural elements of PAR binding to leishmanial ribosomes compared to bacteria (Fig. 2d–h). The delineation of these elements was precluded from earlier X-ray studies performed in minimal RNA-binding pocket constructs^9^ because they reflect only a minimum environment required for drug binding and not the entire chemical milieu of the binding pocket. Difference mapping calculated between the current and the earlier reported structures of the *L. donovani* vacant ribosome^12,13^ also revealed the presence of additional PAR molecules that were found attached to several locations of both the large and small ribosomal subunits (Supplementary Fig. 4). Multiple secondary binding sites were also previously reported in X-ray structures of AGs bound to bacterial ribosomes^14–16^; as these were primarily localized to ribosomal loci with no presumable ribosomal activity, they are mostly regarded as non-specific interactions between the positively charged amino-moieties and the rRNA phosphate backbone. Notably, one PAR molecule was found to bind between H45 of the SSU and H69 of LSU, partially overlapping with the AG secondary binding pocket previously reported in bacterial ribosomes (Supplementary Fig. 4)^15,16^.

### Conserved and non-conserved elements in the binding pocket

The overall architecture of the PAR-binding pocket observed in the cryo-EM structure resembles the previously reported site in bacterial ribosomes where PAR is tightly bound to h44 forcing the flipped-out orientation of the two evolutionary conserved adenine residues A2158 and A2159 (A1492 and A1493 in *E. coli* numbering) (Fig. 2d, Supplementary Fig. 3a). This conformation facilitates direct interactions of the two residues with the 1st and 2nd positions of the mRNA-tRNA codon-anticodon mini-helix at the decoding center and is considered to mediate AG interference with ribosomal decoding. Additional conserved rRNA and rProtein elements crucial for decoding are also shown to penetrate deeply into the PAR-binding pocket. These include the evolutionary conserved helix 69 (H69) of LSU, G626 (G530, *E. coli* numbering) of helix 18 (h18) and a highly conserved loop of protein uS12 (Fig. 2d). These common elements suggest that, similar to bacteria, PAR is most likely to interfere with translational fidelity through stabilization of non-cognate tRNAs at the decoding center by limiting the mobilization of the two conserved adenine residues^17–19^. The secondary binding pocket close to H69, which partially corresponds to the additional pocket found in bacteria (Supplementary Fig. 4), also implies possible interference with translational translocation and ribosome recycling^15,16^.

In addition to the common characteristics of the PAR-binding pocket shared between bacteria and *Leishmania*, two eukaryote specific ribosomal proteins, eS30 and eL41, are shown to be localized within the binding site periphery (Fig. 2e–h). The characterization of these proteins, which have no counterparts in bacterial ribosomes, was precluded from recently reported structures of *Leishmania* and other eukaryotes due to their high mobility in the absence of an A-site tRNA and PAR^13,20,21^. Recent studies in yeast showed that mutations of eS30 and eL41 homologs confer hypersensitivity to AGs^22,23^, suggesting that these proteins may play a role in the organization of the PAR-binding pocket and in eukaryote decoding. These results correlate with our observations that the two proteins maintain tight interactions with several binding pocket elements known to be crucial for decoding (Fig. 2e, f). In addition, as earlier studies in bacteria suggested that mutations in ribosomal proteins localized in proximity to the binding pocket have evolved upon extended exposure to AGs, the two proteins likely serve as hot-spots for parasite resistance development.

eS30 is an essential protein in eukaryotes that is considered to have a function in ribosome biogenesis^24^. While many studies have focused on the role of eS30 in ribosome assembly, its function in the mature ribosome has not been understood. In our *Leishmania* ribosome structure the eS30 N-terminal region penetrates deeply into the decoding center maintaining direct contacts with the PAR-binding pocket at h44 as well as with the anticodon loop of the A-site tRNA (Fig. 2e, f). His5 of eS30, a highly conserved residue within the decoding center (Supplementary Fig. 5), is prone to interact via its imidazole amines with the N1 position of the flipped-out adenine residues as well as with the phosphate backbone of A-site tRNA residue A37 (Fig. 2f). Given that both elements are known to play crucial role in decoding and that these interactions seem to stabilize the A-site geometry upon cognate tRNA binding, we suggest that eS30 is involved in monitoring translational accuracy in eukaryotes. These observations are also in agreement with recent genetic studies in yeast demonstrating that the removal of 23 residues from the eS30 N terminus resulted in increased sensitivity to miscoding agents such as AGs^22^, implying its participation in decoding. The differential sensitivity to PAR that has been reported with these mutants is also largely supported by the current structure where the N terminus of eS30 is shown to interfere with the flipped-out orientation of the two adenine residues, which are also key components facilitating PAR binding.

An additional eukaryote-specific protein that is localized in the PAR-binding pocket is eL41, a single short peptide that forms a eukaryote specific inter-subunit bridge (eB14) (Fig. 2e, Supplementary Fig. 5). The presence of eL41 was unexpected, as previous studies from us and other groups^12,13,25,26^ indicated that the absence of eL41 is a unique characteristic of *Leishmania* and other trypanosomatid ribosomes. Although eL41 ribosomal function is currently not well understood, recent studies in yeast cells deficient in eL41 showed impaired peptidyl transferase activity, reduced translational fidelity and increased sensitivity to AGs^23,27,28^. These observations correlate well with our results, showing that within the SSU, eL41 is localized adjacent to h44 maintaining tight interactions with key elements of the decoding center as well as with several nucleotides that directly interact with PAR.

### RNA modifications reveal an unexpected pattern

**Fig. 3:**
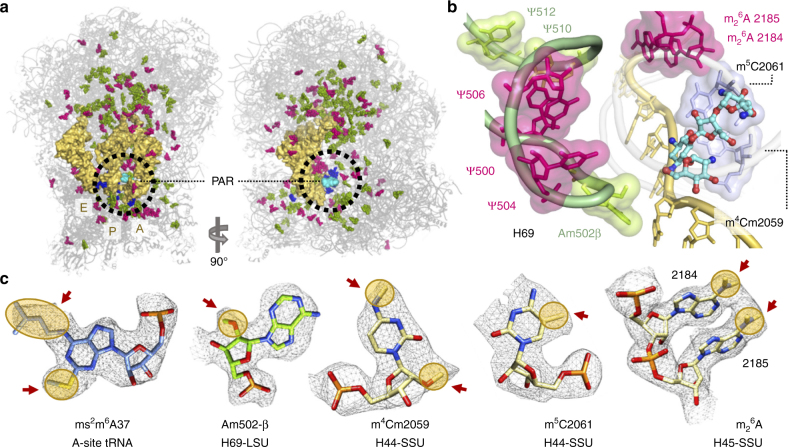
RNA modifications reveal an unexpected pattern of mixed bacterial and eukaryotic origins. **a** The high-resolution structure obtained in this study enabled the direct visualization of rRNA modifications in the leishmanial ribosome, many of which are localized at the decoding center (circled) in close proximity to the PAR binding pocket. 2′-*O*-methylated residues are highlighted in green, pseudouridines in magenta and unique base modifications are colored blue. **b** The PAR-binding pocket at the leishmanial ribosome is decorated with modified residues that are of mixed bacterial and eukaryotic origin. Modifications of bacterial origin (light blue) at h44 of SSU are clustered with two universally conserved modifications at h45 (magenta), to form a bridge that connects elements of the decoding center with mRNA A-site codon (yellow). H69 (light green) of LSU is also heavily modified and penetrates the PAR binding pocket. *Leishmania* H69 modifications of eukaryotic origin are highlighted in green, and universally conserved modifications in magenta. **c** rRNA modifications in the binding pocket. Densities are shown at 3.0 σ. Modified moieties are indicated by arrow and highlighted in yellow

The high resolution of the 91S *Leishmania* ribosome structure enabled the direct visualization of the rRNA modification pattern (Fig. 3, Supplementary Table 3). Such characterization was not possible in earlier reported structures of eukaryotic ribosomes, mostly due to positional variability of the SSU in the absence of PAR and tRNAs. Our structure reveals that the periphery of the PAR pocket is highly enriched with modified rRNA residues (Fig. 3). This observation was anticipated given that the decoding center is also heavily modified in bacteria^29,30^. Unexpectedly however, although *Leishmania* is a eukaryote, some of the rRNA modifications localized to the decoding center are of bacterial origin and have not been affiliated with other eukaryotic divisions. Bacterial specific modifications include m^4^Cm2059 and m^5^C2061 (m^4^Cm1402 and m^5^C1404 in *E. coli* numbering)^29,30^ at h44 (Fig. 3b, c, Supplementary Figs. 7 and 8, Supplementary Table 4). Together with the two universally conserved m^6^_2_A modified residues 2184 and 2185 (1518, 1519 in *E. coli*; 1781, 1782 in yeast; 1850, 1851 in human, Supplementary Figs 7, 8, Supplementary Table 4) of h45^29,31^ these modifications form a cluster that tightly bridges the drug-binding pocket with several elements known to be crucial for decoding (Fig. 3b); these elements include the mRNA-tRNA codon-anticodon mini-helix and the decoding center helices 44 and 45 of SSU and H69 of LSU. Given that the equivalent modifications in bacteria were reported to play an important role in translational fidelity, monitoring of initiation accuracy and susceptibility to several AGs^29^, the *Leishmania* rRNA modifications are positioned to facilitate PAR deleterious activities on parasite translation and growth. Furthermore, the absence of such modifications in yeast and higher eukaryotes might explain the differential selectivity of AGs for *Leishmania* compared to other eukaryotes, which have been shown to be less susceptible to their deleterious effects (Table 1).

In addition to the clustered modification pattern of h44 and h45 of SSU, H69 of the LSU that penetrates the binding pocket is extensively altered with six modifications, three of which are of eukaryotic origin. These include the eukaryote conserved Am502 of chain β (A1913 in *E. coli*) as well as two eukaryote conserved Ψ moieties at positions 510 and 512 (Fig. 3b, c, Supplementary Table 3). These modifications are in addition to the universally conserved Ψ moieties at positions 500, 504, and 506 (correlating to Ψ 1911, 1915, and 1917 in *E. coli*). Given the close proximity of these modifications to the PAR binding pocket, we hypothesize that the local modification pattern of H69 might also be a key determinant for PAR-induced activity in *Leishmania*.

### AGs act on the ribosome by distinct mechanisms

**Fig. 4:**
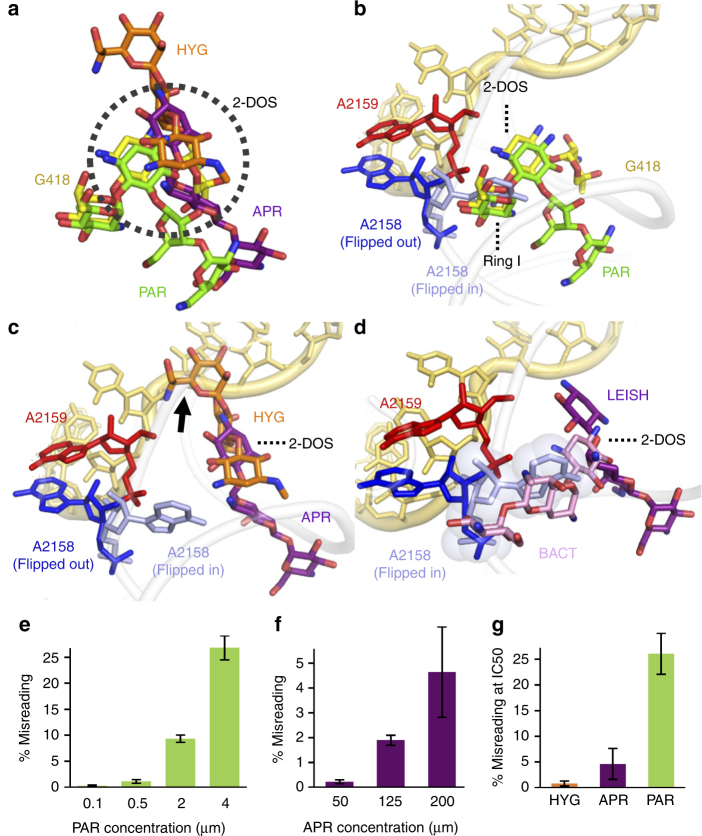
Superposition of AGs bound to the leishmanial ribosome explains their variable interference with decoding. **a** Structures of PAR (green, obtained in this study) and superposed structures of G418 (yellow), HYG (orange) and APR (purple) attached to the leishmanial binding pocket highlight that all derivatives target the binding pocket with the conserved 2-DOS ring serving as an anchor. AGs were docked to the leishmanial binding site by superposing the structures derived from the minimal leishmanial rRNA models for G418 and APR PDB IDs 4k32 and 4k31, respectively, and from the bacterial ribosome structure for HYG, PDB ID 4v64. **b** Di-substituted AGs (PAR, NEO, GEN and G418) bind the leishmanial ribosome in a similar fashion, with rings I and II (2-DOS) serving as anchors forcing the flipped out orientation of A2158-A2159 (NEO and GEN were removed from the figure for simplicity as they only differ from PAR and GEN by the identity of their 6′-position). **c** Mono-substituted AGs (APR and HYG), lacking ring I, anchor the ribosome through their 2-DOS ring, with APR rings III–V directed toward the internal helical core and HYG substituent at position 5 rotated by 180°, thus penetrating the mRNA accommodation channel and clashing with the mRNA at the P-site (clash is indicated by a black arrow). Both APR and HYG lack ring I and thus do not dictate the flipped-out conformation of A2158 (flipped-in conformation is highlighted in light blue indicating no clash with the drugs). **d** Superposition of APR bound to *Leishmania* ribosome (LEISH, purple, PDB 4k31) and bacterial ribosomes (BACT, light pink, PDB 4aqy) indicating a 180° rotation in the bacterial binding. Such discrepancy in binding dogma results from sequence alternations in the binding pocket and indicates that while in bacteria APR clashes with the flipped-in conformation of A2158, thus forcing it’s flipped-out orientation, no such interference occurs in *Leishmania*, potentially explaining the poor potency when compared to bacteria. **e**, **f** In vitro incorporation of tRNA at UGA stop codon locations by exposure to increasing PAR (**e**) and APR (**f**) concentrations. **g** Misreading events as measured by UGA stop codon read-through at 50% inhibition (IC_50_ values). Each value represents the mean ± standard error of at least three independent experiments. Calculated IC_50_ values are highlighted in Table 1

Our biochemical results on the activity of AG derivatives against *Leishmania* (Fig. 1b) generated a pattern of differential activity that did not correlate with their ability to inhibit bacterial translation nor cytosolic translation in higher eukaryotes (Table 1). The lack of correlation with inhibition of bacterial translation further supports the notion that the mitochondrial ribosome, which shares greater similarity with the bacterial translation apparatus, is not the main target of AGs in *Leishmania*. In addition, our findings regarding species selectivity may be of high value for the future development of anti-leishmanial derivatives. To further understand the molecular attributes of AG structure–function divergence in *Leishmania* we used a combination of biochemical assays designed to evaluate AG interference with leishmanial decoding coupled with an in silico investigation of the differentiated derivatives within the binding pocket (Fig. 4). The results suggest that all AGs bind to the leishmanial ribosome in roughly the same location with their conserved 2-deoxystreptamine (2-DOS) ring serving as a main anchor (Fig. 4a). Nevertheless, as the non-conserved rings variably distribute around it, they are not expected to induce the same structural rearrangement of the binding pocket and are likely to act by different mechanisms.

The di-substituted derivatives PAR, G418, GEN and NEO, which share rings I and II (2-DOS), are similarly anchored to the pocket core with ring I, dictating the flipped-out conformation of the two conserved adenine residues A2158-2159 (Fig. 4b). These binding characteristics indicate a similar mechanism of action for these derivatives and imply interference with translation fidelity and ribosome decoding. These observations also correlate with our in-vitro results indicating that upon introduction of increased PAR concentrations the rate of mismatched tRNA incorporation at the stop position was significantly elevated (Fig. 4e), thus implying strong interference with leishmanial decoding. The superior inhibition of translation observed for the 6′-hydroxy derivatives PAR and G418 compared to their 6′-amino analogs NEO and GEN (Fig. 1b, Table 1) seems to mostly depend on the identity of the 6′ moiety of ring I dictating the interaction of the eukaryote conserved G2065 (A1408 in *E. coli*). These observations are consistent with earlier studies in eukaryotes, as well as with mutagenesis studies in bacteria, demonstrating how A1408G substitution confers resistance to 6′-amino containing residues^9,32,33^.

As opposed to the di-substituted derivatives, the mono-substituted APR and HYG are differently docked within the binding pocket, and in the absence of ring I, they seem to interact only with the phosphate backbone of A2159 (A1493 in *E. coli*) but not with A2158 (A1492) (Fig. 4c). Indeed, our results indicate that HYG does not induce misreading of the genetic code even at concentrations corresponding to its IC_50_ value (Fig. 4g). These results are also in agreement with earlier reported studies in bacteria that indicate negligible miscoding activity for HYG^34,35^. Surprisingly, APR showed increased misreading activity at elevated concentrations (Fig. 4f), a finding that does not correlate with APR inability to interfere with the mobility of A2158, and is also in contradiction to recent reports indicating the lack of misreading for APR in bacteria^36^. Nevertheless, when compared to PAR, APR misreading activities are almost negligible (Fig. 4g, misreading activities at IC_50_ levels for PAR and APR are 26% and 5%, respectively). APR and HYG effects on leishmanial translation differ by four orders of magnitude, with HYG acting vigorously at low nanomolar concentrations and APR causing only mild inhibitory effects at high micromolar concentrations (Fig. 1b, Table 1). These discrepancies in potency may be explained by their orientation within the binding pocket, which shows that apart from the co-localization of their 2-DOS rings, their substituent rings are directed 180° away from each other (Fig. 4c). As a result, while APR substituent rings are only attached to h44 with no inducible effect on the helical conformation, HYG is penetrating the mRNA binding channel and is therefore prone to interfere with the P-site mRNA codon. The suggested divergence in interactions may explain why APR has negligible effect on leishmanial translation whereas HYG is a potent inhibitor of translation. Notably, the distinct inhibition pattern of HYG and APR in *Leishmania* does not correlate with their activities in bacteria where they both inhibit translation at similar concentrations (Table 1). Such species related discrepancies in ribosome inhibition patterns could be explained by the observation that HYG interacts with binding pocket nucleotides that are shared between *Leishmania* and bacteria (Supplementary Fig. 6). Thus, HYG is predicted to bind similarly to the two binding pockets while APR, which also interacts with non-conserved residues, is differently bound to the leishmanial binding pocket when compared to bacteria (Fig. 4d, Supplementary Fig. 6)^8^. As a result, while HYG inhibits both ribosome species at similar concentrations, APR shows a 4 orders of magnitude reduced activity in *Leishmania* (Table 1). Such differential selectivity for bacteria is further supported by differences in re-organization of the binding pocket upon APR attachment to the distinct ribosome species^8^ (Fig. 4d). APR bound to the bacterial ribosome induces a steric clash with the intra-helical conformation of A1493 (2059 in *Leishmania*), thus forcing its flipped out conformation in a way that is similar to other AGs. The bound conformation in *Leishmania* does not induce such a conformational change. The lack of HYG binding selectivity for the different microorganisms (Table 1, Supplementary Fig. 6) also indicates why HYG can’t be used clinically to treat leishmaniasis or bacterial infections, where it inhibits the eukaryotic ribosome at similar therapeutic concentrations, thereby exhibiting increased toxicity^35^.

Taken together, the biochemical and structural data presented here indicate that AGs mediate their activity against *Leishmania* by interfering primarily with cytosolic translation, and that their induced activities in these eukaryotic parasites is highly differentiated when compared to bacteria and higher eukaryotes. These findings highlight a selectivity window that can be used for the development of species-specific derivatives for the treatment of parasitic infections.

## Methods

### In vitro inhibition of translation assays

In order to assess cytosolic ribosome susceptibility and selectivity to putative anti-leishmanial compounds, we performed three different cell-free transcription-translation assays in S30 extracts of *E. coli* (Promega), rabbit reticulocytes (Promega) and *L. tarentolae* (Jena Bioscience). *L. tarentolae* is a non-pathogenic *Leishmania* strain that does not cause disease in human. The ribosomes derived from this species are highly similar to *L. donovani* ribosomes. Three plasmids, compatible with each extract system, were used in this study: pBEST*luc*TM (Promega) for the prokaryotic translation assay, Luciferase T7 DNA (Promega) for the reticulocytes and pLEXSY-*invitro*2-EGFP (Jena Bioscience) for *Leishmania*. Firefly luciferase was used as a reporter gene for the bacterial and reticulocyte systems and EGFP for *Leishmania*. Reaction mixtures were prepared as suggested by the manufacturer except that the final reaction volume was adjusted to 15 μl to which 1.5 μl 10× of the relevant compound concentration were supplemented. Assays were performed in white polystyrene 96-well flat bottom plates (Nunc) for the luciferase and black polystyrene 384-well flat-bottom plates (Greiner) for the EGFP. Incubation times were 60 min at 37 °C, 90 min at 30 °C and 120 min at 26 °C for the bacterial, reticulocyte and *Leishmania* systems, respectively. Reactions were stopped by quick snap cooling followed by a 5-min incubation on ice. Luciferase activity was measured for each well following the addition of 50 μl Luciferase Assay reagent (Promega) by recording the chemi-luminescence signal on supplemented with automatic reagent injector (Tecan). EGFP fluorescence (*λ*_ex_ = 488 nm; *λ*_em_ = 507 nm) was recorded on Tecan Infinite R®F200 microplate reader (Tecan). Extracts lacking the circular DNA template were used as negative control to calculate the fluorescence*/*chemi-luminescence background. Reaction mixtures without compound presence were used as positive control and were regarded as 100% translation. At least six different concentrations were used to plot each translation inhibition curve, experiments were performed in three independent repeats in duplicates. Half-maximal inhibition concentration (IC_50_) values were calculated from the concentration–response fitting curves of using GraFit7 software^37^.

### In vitro assessment of misreading

To examine AG modes of action in *Leishmania*, we designed a dual-reporter assay whereby an in-frame linker containing a UGA stop mutation was introduced in between two genes encoding Renilla and Firefly luciferases cloned into an in vitro* Leishmania* expression system. A similar system was previously used to assess stop codon read-through events in bacteria as well as other eukaryotes, and as these levels seemed to correspond to tRNA misreading levels it has also been previously used to evaluate infidelity of translation^38,39^.

The content of a p2luc dual-luciferase derived from an SV-40 vector with either wild type or an in-frame TGA stop codon poly-linker was cloned into pLEXSY-*invitro*2 that is compatible with *Leishmania* in vitro translation (Jena Bioscience). SV-40 constructs were a generous gift by Professor Timor Baasov. Cloning has been performed by restriction-free (RF) PCR methodology as previously described by Unger et al.^40^.

5′-TGTAAAGACATTAAACACGTAAGTGAAACCATGACTTCGAAAGTTTATGATCCAG-3′ and 5′-CCGGGGATCCTCTAGAGGTCGACAAGCTTGTTACAATTTGGACTTTCCGCCCTTC-3′ were used as forward and reverse primers, respectively. Primers were produced by custom design (Sigma). The in vitro misreading assays were performed in *L. tarentolae* lysates and were designed similarly to the inhibition of translation assay reported herein. The main modifications were that the final reaction volume was adjusted to 20 μl and that the assay has been performed in white polystyrene 96-well flatbottom plates (Nunc). Luciferase activity was determined after 120 min incubation at 26 °C, using the Dual luciferase reporter assay system (Promega) and was performed according to the manufacturer’s instructions.

### *Leishmania* promastigote viability assays

*Leishmania* susceptibility assays were performed using promastigotes of *L. donovani* MHOM/ET/2009/GR356 clone IV. Parasites were grown in complete M199 medium (Sigma) containing 20% FCS at 26 °C. LC_50_ was determined by serial dilution of tested compounds in complete promastigote medium. Compounds were aliquoted in triplicates (125 μl per well) to 96-well flat-bottom plates (Nunc). Promastigotes (2.0 × 10^6^ cells per ml; 125 μl per well) were added to each well and incubated for 72 h at 26 °C. The alamarBlue (AbD, Serotec) viability indicator was added (25 μl per well) and the plates were incubated for 5 h. Fluorescence (*λ*_ex_ = 544 nm; *λ*_em_ = 590 nm) was measured in a microplate reader (Fluoroskan Ascent FL). Complete medium was used as a negative control (0% inhibition of promastigote growth). Amphotericin B (1 μM), a drug used to treat VL, was included as a positive control in each plate.

### Ribosome purification and complex formation

*L. donovani* (MHOM/ET/2009/GR356 clone IV) promastigote growth and 91S ribosome purification has been performed as previously described^12^. Ribosome complexes with mRNA, three tRNA molecules and paromomycin were assembled by sequential addition of an mRNA fragment (**CACC** AUGUUCAAA, GE Dharmacon) containing the leishmanial Kozak sequence^41^ (highlighted in bold) a P-site start codon (AUG, underlined) and an A-site Phe codon (UUC), P-site tRNA_fmet_ (*E.coli*, Sigma), A-site tRNA_phe_ (*E.coli*, Sigma) and Paromomycin sulfate (Sigma) at 1:100:5:5:100 stoichiometric ratio. Complex assembly has been performed at 26 °C in ribosome conservation buffer (20 mM HEPES–KOH pH 7.6, 100 mM KOAc; 10 mM Mg(OAc)_2_, 10 mM NH_4_OAc, 2 mM β-mercaptoethanol and 1:40 dilution of RNAsin U (Promega)) with relaxation times of 30 min after the addition of each complex component. Ribosome final concentration was 125 nM.

### Cryo-EM data acquisition

A volume of 3.5 μl of ribosome complex samples was applied on glow-discharged holey carbon grids (Quantifoil R2/2, 200 mesh) coated with a continuous thin carbon film. The grids were blotted and plunge-frozen using a Vitrobot Mark IV (FEI Company). Cryo-EM micrographs were recorded at liquid nitrogen temperature on a Titan Krios electron microscope (FEI) operating at 300 kV. Micrographs were recorded at a nominal magnification of ×29,000 using a K2 Summit direct electron detector (Gatan, Inc.) using a pixel size of 1.02 Å/pixel, with a dose rate of ~5.0 electrons/Å^2^/s and defocus values ranging from −0.9 to −1.8 μm. The total exposure time was 6.0 s and intermediate frames were recorded in 0.1 s intervals resulting in an accumulated dose of ~30 electrons per Å^2^ and a total of 60 frames per micrograph.

### Cryo-EM image processing and 3D reconstructions

Cryo-EM images were subjected to motion correction using MotionCorr2^42^. A sum of all frames, filtered according to exposure dose, in each image stack was used for further processing. CTF parameters for each micrograph were determined by CTFFIND4^43^. Particle selection, two-dimensional and three-dimensional classifications were performed in RELION 1.4^44^ as previously described^12^. The resulting particle projections were subjected to further refinement with alignment focusing on the SSU and LSU, respectively. Reported resolutions are based on the gold-standard Fourier shell correlation (FSC) using the 0.143 criterion (Supplementary Fig. 2). Local resolution was determined using ResMap^45^ with half-reconstructions as input maps (Supplementary Fig. 2).

### Model building and refinement

Model building of the ribosomal LSU has been performed by fitting the previously reported model for *L. donovani* LSU (PDB ID 3JCS) to the calculated density map using Chimera^46^. The docked model was manually manipulated using COOT^47^ real-space and geometry restraint commands to fit into the density maps. De-novo sequence guided model building was applied to additional features that were better resolved in the current maps. SSU model building has been performed by template guided use of docked human and yeast ribosome structures (PDB IDs 4UG0 and 4V88, respectively) as previously described for LSU model building in *L. donovani* LSU^12^. The rRNA and protein sequences used for modeling were extracted from the *L. donovani* GR356 whole-genome sequence that was annotated based on the *L. infantum* (JPCM5) and *L. donovani* (BPK282A1) genomes deposited at TriTrypDB^48^. Protein content was also examined by MS analysis (Supplementary Table 2 **)**. PAR molecules were manually docked to unassigned densities that clearly demonstrated the shape of an intact four ringed structure. The docked structures were later real space refined in COOT to better fit the electron density and refined by Phenix^49^. Model refinement was performed by combination of PHENIX and COOT as previously described^12^. Structure validation was done by using Molprobity^50^. Model overfitting was evaluated through its refinement against cryo-EM half maps (Supplementary Fig. 2). Figures were created using PyMol^51^, and the UCSF Chimera package^46^. Local resolution plots were generated in ResMap^45^.

### Liquid chromatography-coupled mass spectrometry analysis

LC-MS analysis to determine samples purity and protein content has been performed as previously described^12^. LC-MS analysis of *L. donovani* 18S rRNA was performed as follows: The 18S rRNA was extracted from 91S *L. donovani*-purified ribosome sample with phenol/chloroform and was further purified by reversed phase chromatography on a PLRP-S 300 Å column (2.0 mmID × 100 mmL, 3 μm particle size, Agilent Technologies). rRNA elution was performed with a 120-min linear gradient of 11.2–16.8% (v/v) acetonitrile in 100 mM triethylammonium (TEAA) acetate buffer, pH 7.0, containing 0.1 mM diammonium phosphate at a flow rate of 50 µl/min at 60 °C^52^. The purified 18 S rRNA (~100 fmol) was digested with RNase T1 (3 ng/µl, Worthington) in 100 mM TEAA buffer (pH 7.0) at 37 °C for 60 min and the digests were analyzed by direct nanoflow LC-MS system equipped with a spray-tip column (150 μmID × 120 mmL) packed with a reversed phase material (Develosil C30-UG-3, 3 μm particle size; Nomura Chemical) connected to a high-resolution mass spectrometer (Q Exactive Plus, Thermo Fisher Scientific) through an electrospray interface as previously described^53–55^. Briefly, the eluate from the spray-tip column was introduced at –1.4 kV with the aid of a spray-assisting device^54^ into a mass spectrometer operating under the negative ion mode and the MS and tandem MS (MS^2^) spectra were acquired in the data-dependent mode to automatically switch between MS and MS^2^ with a mass resolution of 35,000 and17,500 at *m*/*z* 200, respectively. The resulting spectra were analyzed to assign to the *L. donovani* 18S rRNA sequences and to identify the post-transcriptional modifications with the genome-oriented database searching software Ariadne (http://ariadne.riken.jp/)^56^ under the following search parameters: the maximum number of missed cleavages was set to 1; the variable modification parameters included two methylations per RNA fragment for any residue; RNA mass tolerance of ±5 ppm and MS/MS tolerance of ±20 ppm were allowed. When a methylated oligonucleotide was identified, the MS/MS spectrum was inspected to distinguish the methyl group attached to the base or sugar by the presence or absence of methylated base loss from the parent ion at *m*/*z* 225.02 used as a signature of 2′-*O*-methylated nucleotide.

### Data availability

The cryo-EM data have been deposited in Electron Microscopy Data Bank (EMDB) under accession codes EMD-7024, EMD-7025 for the SSU and LSU. The atomic model has been deposited in the Protein Data Bank (PDB), ID numbers 6AZ1 and 6AZ3 for SSU and LSU, respectively. The data that support the findings of this study are available from the corresponding author upon request.

## Electronic supplementary material

Supplementary Information
