# Supplementary material for: Atomic resolution snapshot of Leishmania ribosome inhibition by the aminoglycoside paromomycin
Source: Nat Commun. 2017 Nov 17;8:1589. doi: 10.1038/s41467-017-01664-4 (PMC5693986; doi:10.1038/s41467-017-01664-4)
Supplement: Supplementary file 1 — Supplementary Information [file 41467_2017_1664_MOESM1_ESM.pdf]

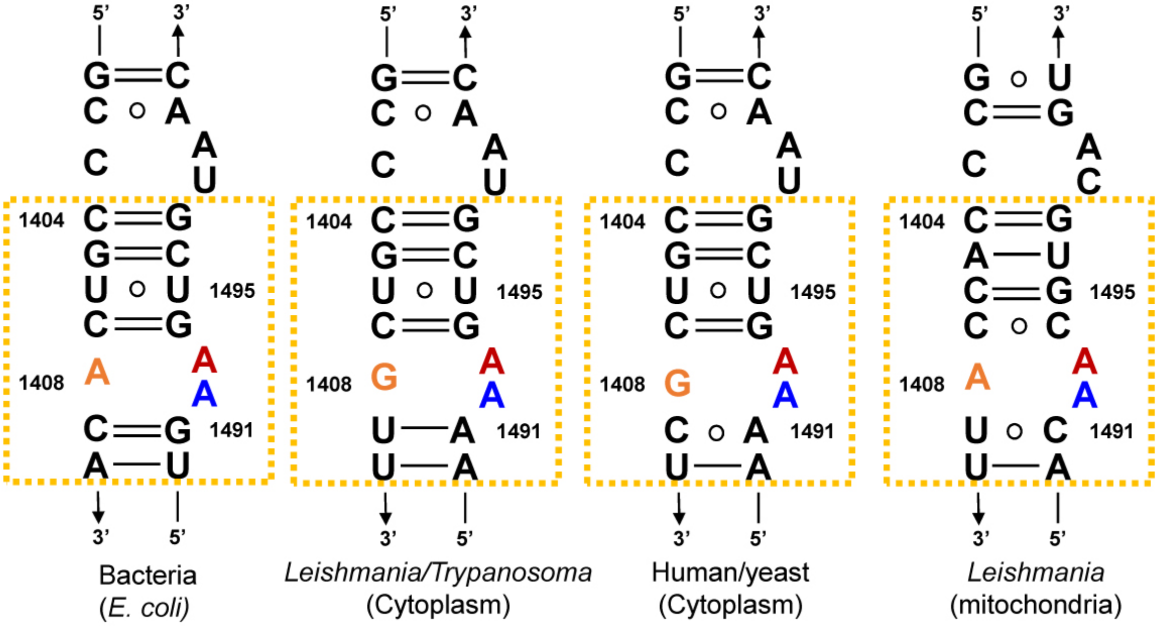

Supplementary Figure 1 | 2D representations of AGs binding pockets.

Secondary structures of AG binding pockets in bacteria, *Leishmania* and human cytosolic and mitochondrial ribosome decoding centers. The leishmanial mitochondrion has been highlighted as the main target of AGs in the parasitic cell primarily due to the common identity of the nucleotide residue at position 1408 (*E. coli* numbering) to bacteria. The universally conserved adenine residues 1492 and 1493, also known to mediate AG activities in bacteria, are marked in blue and red, respectively, and the non-conserved G or A at position 1408 in orange.

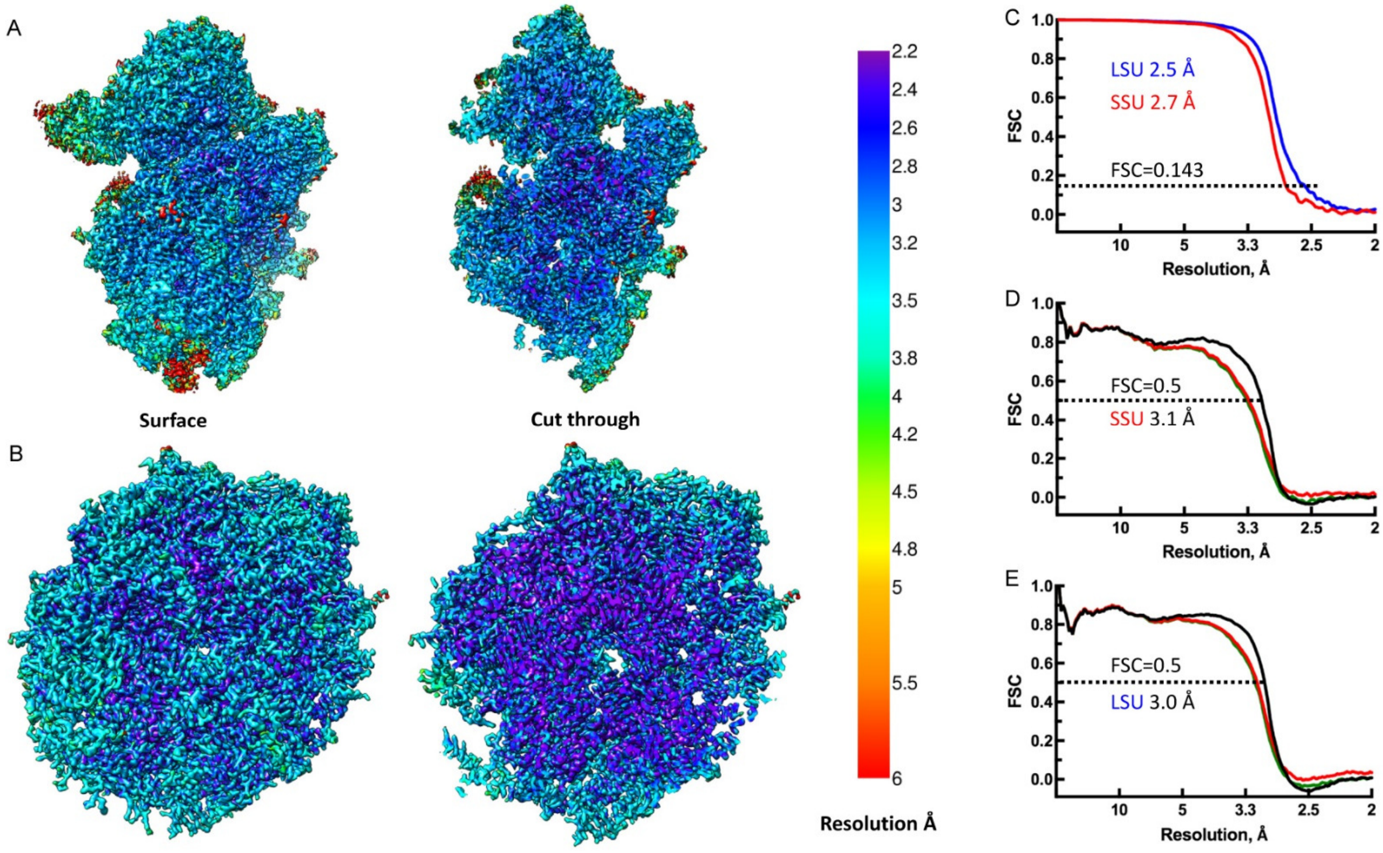

Supplementary Figure 2 | Local map resolution and FSC curves.

Surface (left) and cross-section (right) rendering of the cryo-EM density maps colored according to local resolution of SSU (**A**) and LSU (**B**). (**C**) **"**Gold standard" FSC curves for the final 3D map of SSU and LSU indicates nominal resolutions at 2.7 Å and 2.5 Å (FSC=0.143 criterion), respectively. Evaluation of model-map correlation for SSU (**D**) and LSU (**E**). FSC curves of the final refined model versus the final cryo-EM maps (full dataset, black), of the outcome of model refinement with a half map versus the same map (red), and of the outcome of model refinement with a half map versus the other half map (green). The excellent agreement between red and green curves suggests lack of overfitting.

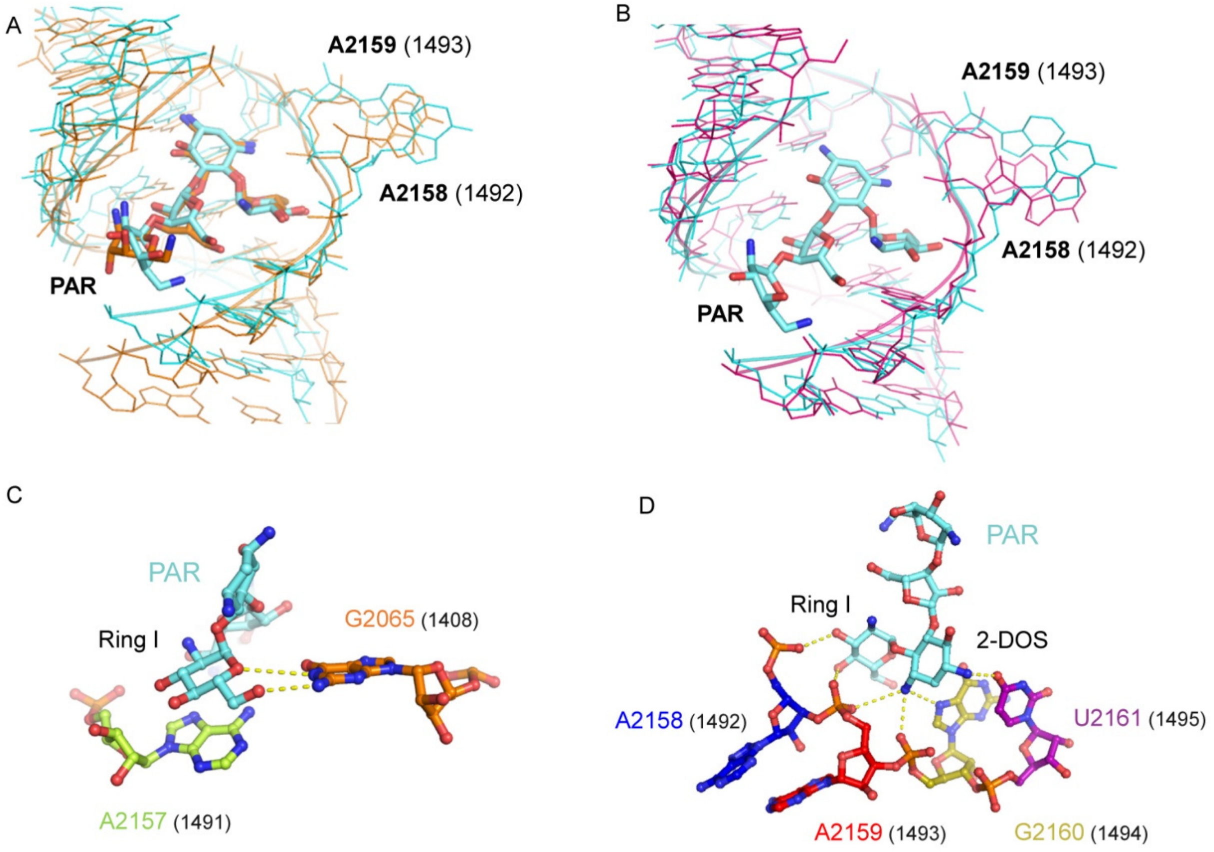

Supplementary Figure 3 | AG minimal binding pockets in bacteria and *Leishmania*

A. Superposition of PAR bound to the bacterial (orange) and leishmanial (cyan) ribosomes shows the similarity between the binding sites and drug binding patterns. PDB IDs are 1J7T and 6AZ1, respectively. **B.** Comparison between the cryo-EM structure (cyan) with the X-ray structure of the minimal binding site (pink). PDB IDs are 6AZ1 and 4ZC7, for the cryo-EM and minimal X-ray structures, respectively. (**C**-**D**) PAR targets h44 by a complex ensemble of electrostatic interactions where rings I and II, also common to other AG family members, act as a stable anchoring scaffold driving the conformational reorganization of the binding pocket. **C.** Ring I mainly interacts with non-conserved RNA residues by forming a pseudo base pair with the eukaryote specific G2065 (A1408 in *E. coli*) while stacking upon the non-conserved A2157 (G1491 in *E. coli*). **D.** The 2-deoxystreptamine (2-DOS) ring contacts the two evolutionary conserved A2158 and A2159 via their phosphate backbone while also interfering with the highly conserved residues G2160 and U2161 (1494-5 in *E.coli*).

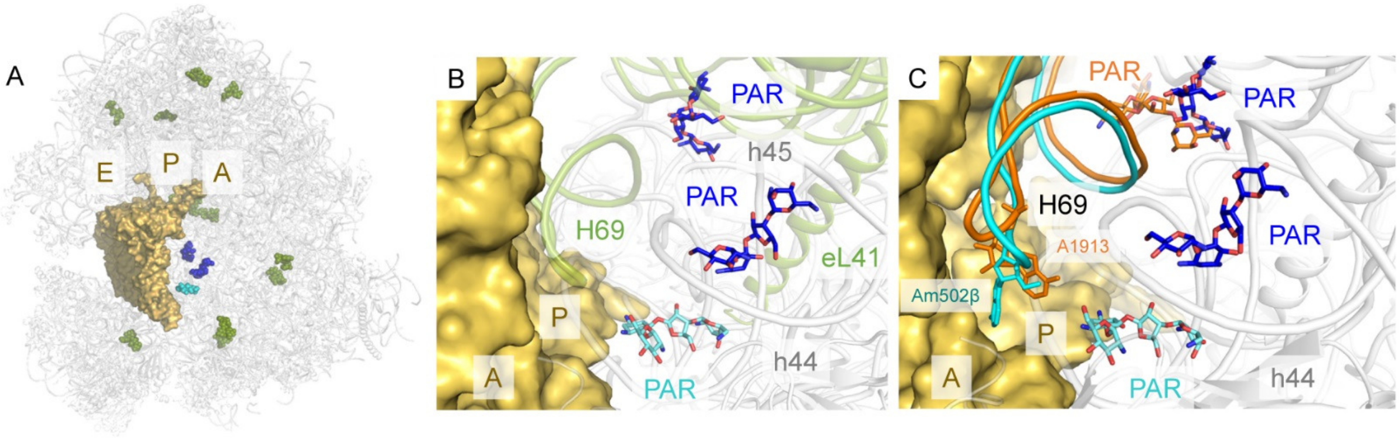

Supplementary Figure 4 | PAR binds to multiple secondary sites on the leishmanial ribosome

A. An overall view of PAR primary (cyan) and secondary binding pockets in the leishmanial ribosome. Molecules that are in close proximity to the decoding and initiation centers are highlighted in dark blue. Molecules that are bound in non-functional regions are shown in green. These observations are consistent with previously reported crystallographic studies that indicated the presence of alternative binding sites of AGs within the large [1,2] and small[3] subunits of bacterial ribosomes (with 6 and 8 moieties bound for PAR and APR structures, respectively). Since most of these non-canonical binding sites are localized at ribosomal locations that are not in proximity to any known functional sites, they are mostly regarded as non-specific electrostatic interactions between the positively charged amino moieties of PAR and the negatively charged phosphates of the rRNA. **B.** As opposed to the randomly scattered PAR molecules, two of the non-canonical sites are localized in proximity to the decoding center, one further down at h44 and a second between h45 of SSU and H69 of the LSU. **C.** The secondary binding pocket at leishmanial H69 (blue) partially correlates with the alternative pocket also found in bacterial H69 (orange) [1,2].

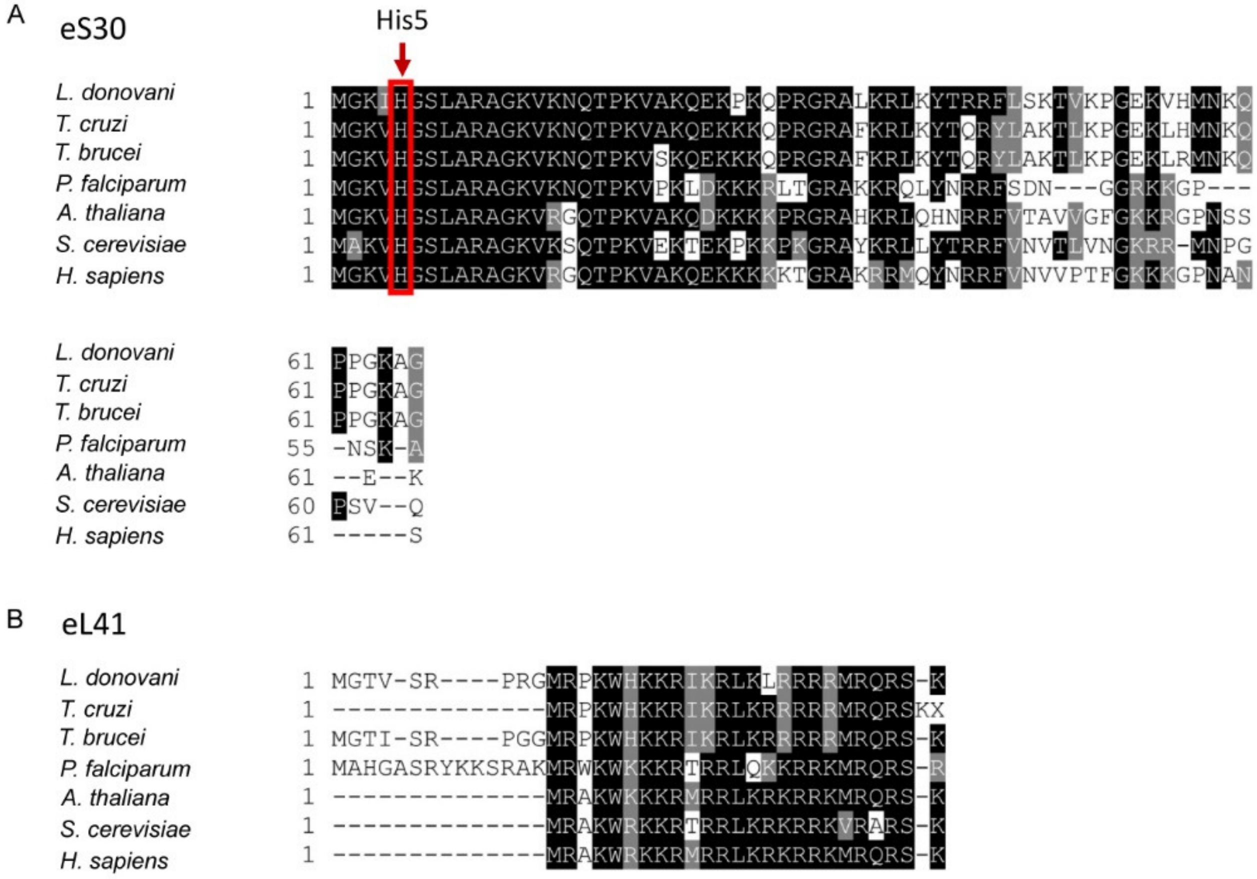

Supplementary Figure 5 | Eukaryote specific proteins in the PAR binding pocket.

A. Multiple alignment of the eukaryote specific eS30 indicates high eukaryote conservation with His5 highlighted as an evolutionary conserved residue. **B.** eL41, which was previously reported to be missing in *Leishmania*, was clearly observed in the EM density maps of the present study. A through genome search indicates the presence of eL41 in the leishmanial genome in three copies. Multiple alignment with sequences derived from previously reported eukaryotes indicates high conservation with N-terminal extensions for the parasites *L. donovani*, *T. brucei* and *P. falciparum*.

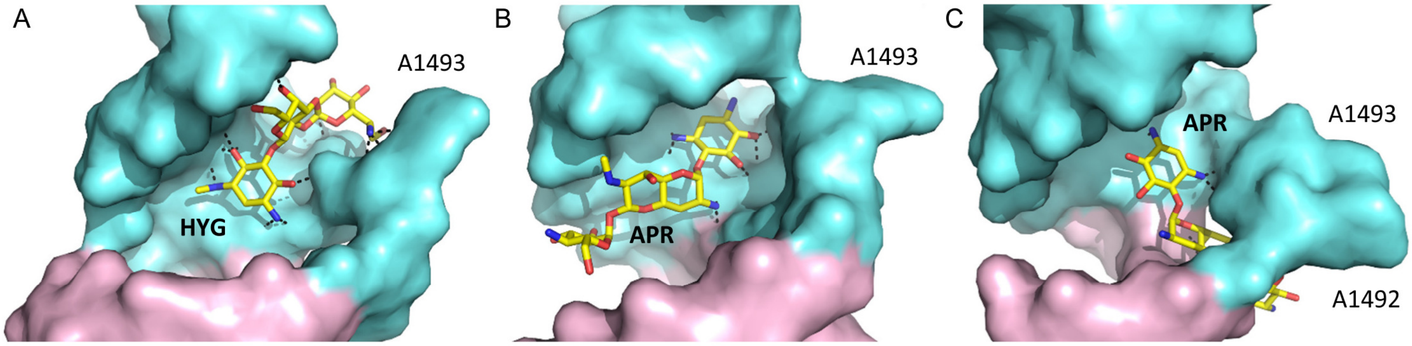

Supplementary Figure 6 | Interaction of APR and HYG with different ribosomal species

HYG (**A**) and APR (**B**-**C**) bound to their ribosomal binding sites. **A.** HYG bound to bacterial ribosome decoding center. APR binding to *Leishmania* ribosome (**B**) and bacterial ribosome (**C**) indicates differential interaction with the binding pocket based on interactions with non-conserved residues. HYG and APR are represented as sticks and the ribosomal surface is shown in surface representation. The surface colors indicate residue conservation among prokaryotes and eukaryotes, where residues marked in light blue are highly conserved and residues marked in light pink are highly diverged. Potential hydrogen bonds are indicated in dashed black lines. PDB accession codes for HYG-bacterial complex, APR-leishmanial complex and APR-bacterial complex are 4V64, 4K31 and 4AQY, respectively. Nucleotide numbers of mobile adenine residues are highlighted in black and are numbered according to their common nomenclature in *E. coli*.

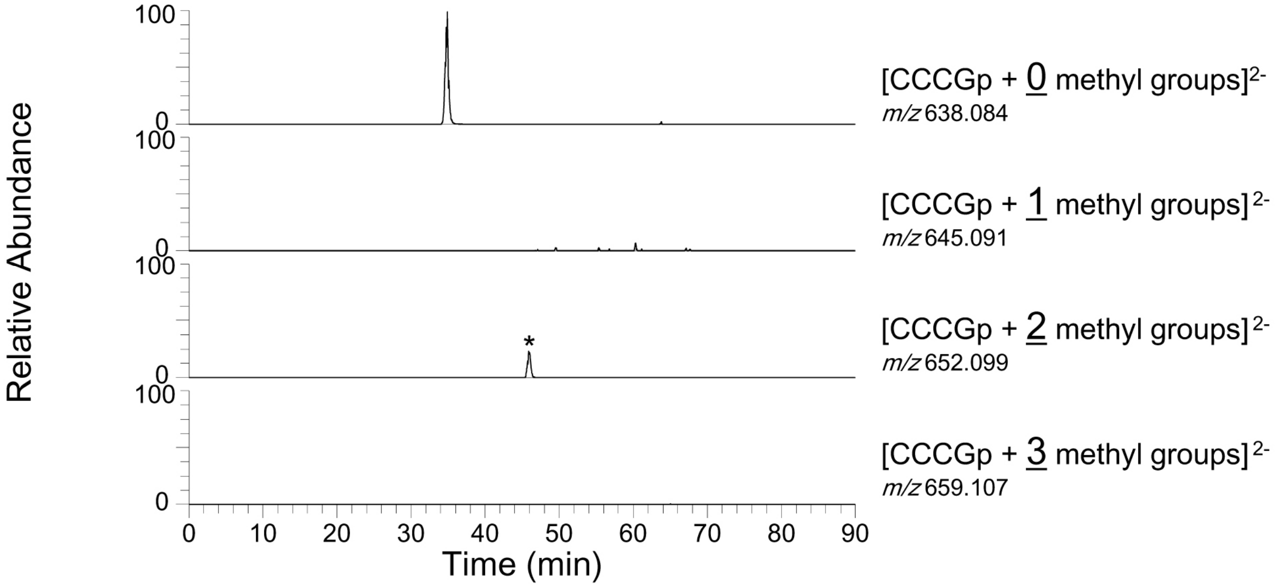

Supplementary Figure 7 | Extracted ion chromatogram (EIC) of the fragments containing CCCGp sequence produced by the RNase T1 digestion of *L. donovani* 18S rRNA.

The signal height of the dimethylated CCCGp+2 methyl groups (marked with asterisk) was approximately one-fourth that of CCCGp+0 methyl group, suggesting that RNase T1 digest of 18S rRNA contained one dimethylated and three unmethylated CCCGp that could be produced from 4 CCCGp sequences at positions 755-758, 1345-1348, 2059-2062, and 2119-2122. Note that the digest contained no trimethylated CCCGp. The MS^2 spectrum of the signal with asterisk is shown in **Supplementary Figure S8**. The experimental details are provided in Experimental procedures. The sequence, methyl group, and m/z value of CCCGp are indicated to the right. The most intense signal (CCCGp + 0 methyl groups) was set to a relative intensity of 100%, and the peaks in the other spectra were scaled accordingly.

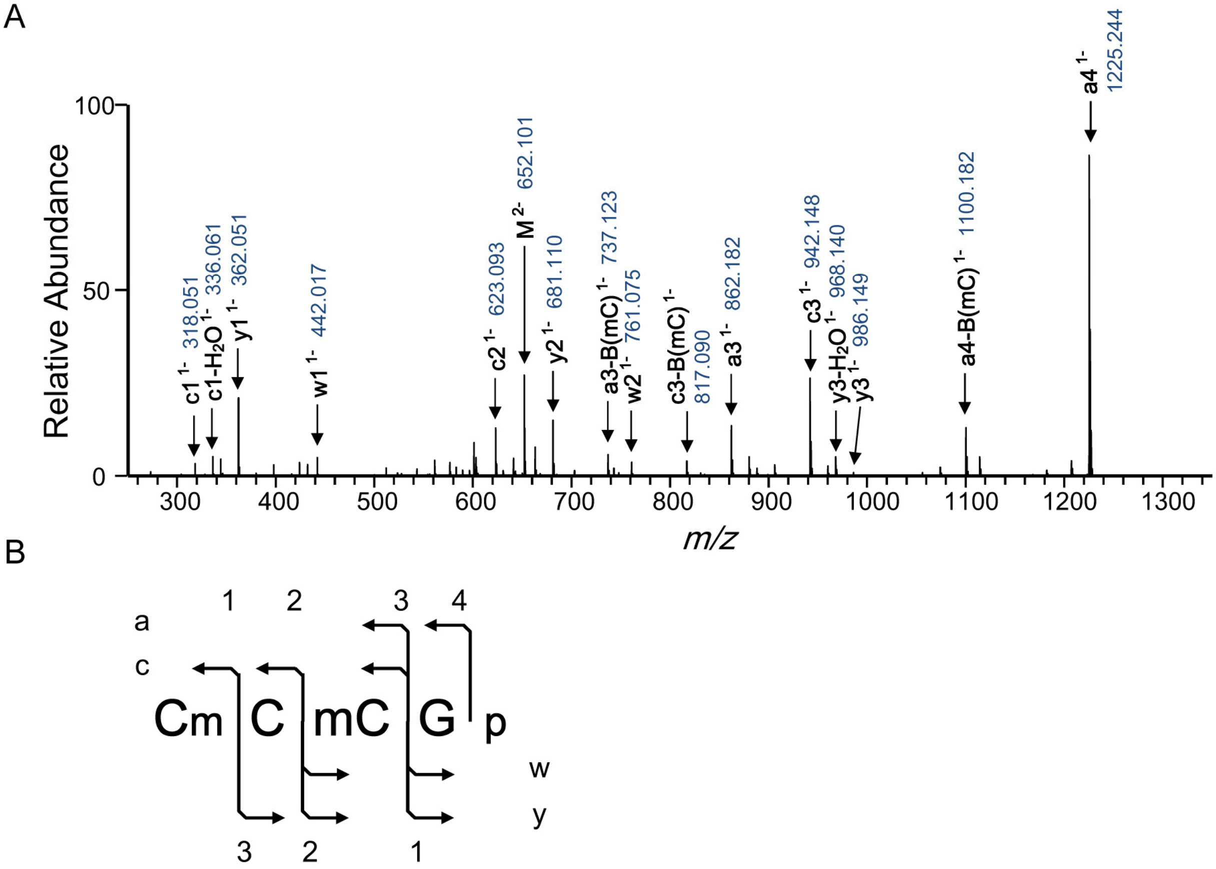

Supplementary Figure 8 | Determination of the methyl position in the dimethylated CCCGp fragment.

A. Collision induced dissociation spectrum of the dimethylated CCCGp in **Supplementary Figure S7**. The major sequencing ions are indicated with the precise m/z values. M, precursor molecule; -B(mC), a fragment ion lacking methylated cytosine. **B.** Sequence of the dimethylated CCCGp determined by the MS^2 spectrum.

Supplementary Table 1| Cryo-EM data collection and model refinement

**Data collection**		
Particles	141,028	
Pixel size (Å)	1.02	
Defocus range	-0.8 to -1.9	
Voltage (kV)	300	
Electron dose (e^{-}/A^{2})	30	
**Refinement**	SSU	LSU
CC map_model	0.7772	0.7910
**Model content**		
Total number of atoms	80,889	126,595
No. of rRNA chains	5	8
No. of protein chains	33	42
No. of ligands	6	10
No. of solvent molecules	288	1055
**Model quality**	SSU	LSU
**RMSD**		
Bond lengths (Å) / Bond angles (°)	0.01/1.166	0.009/1.091
**Ramachandran plot statistics**		
Most favored (%)	93.91	93.33
Outliers (%)	0.08	0.17
**Rotamer outliers (%)**	1.30	1.01
**C-beta deviations**	0	0
**RNA validation**		
Correct sugar puckers (%)	98.45	98.44
Good backbone conformations (%)	69.37	72.39
**Clashescore**	4.40	4.55
**B_iso_{mean}**	61.4	54.8

Supplementary Table 2| Proteins and rRNA compositions for SSU[a] and LSU[b]

a.

Universal name	Chain ID	Length	Modeled range	TritrypDB ID	MS score (Mascot)
18S	1	2203 b	1-2203	LdBPK_27rRNA6	-
A- tRNA	2	76 b	1-76	-	-
P- tRNA	3	76 b	1-76	-	-
E- tRNA	4	76 b	1-76	-	-
mRNA	5	13 b	1-12	-	-
eS1	A	264 aa	22-246	LdBPK_350410.1	3881.33
uS2	B	246 aa	1-211	LdBPK_365240.1	323.07
uS3	C	219 aa	2-213	LdBPK_330960.1	3250.67
uS4	D	190 aa	1-182	LdBPK_361310.1	1513.88
eS4	E	273 aa	2-261	LdBPK_131120.1	4960.24
uS5	F	265 aa	44-263	LdBPK_320460.1	4183.04
eS6	G	249 aa	1-238	LdBPK_151530.1	59.95
uS7	H	190 aa	2-190	LdBPK_110960.1	802.47
eS7	I	200 aa	1-200	LdBPK_010440.1	1349.30
uS8	J	130 aa	2-130	LdBPK_111180.1	971.14
eS8	K	220 aa	2-216	LdBPK_242160.1	1392.02
uS9	L	149 aa	7-149	LdBPK_260840.1	-
uS10	M	116 aa	14-115	LdBPK_281100.1	-
eS10	N	153 aa	32-122	LdBPK_361050.1	1528.40
uS11	O	144 aa	8-144	LdBPK_281050.1	1191.47
uS12	P	143 aa	1-142	LdBPK_211300.1	826.25
eS12	Q	141 aa	27-141	LdBPK_130460.1	2096.50
uS13	R	153 aa	2-143	LdBPK_360990.1	1088.92
uS14	S	57 aa	4-57	LdBPK_282650.1	239.69
uS15	T	151 aa	2-143	LdBPK_333300.1	1170.22
uS17	U	173 aa	5-160	LdBPK_211790.1	121.31
eS17	V	143 aa	2-123	LdBPK_282750.1	-
uS19	W	152 aa	22-135	LdBPK_220340.1	229.15
eS19	X	179 aa	25-176	LdBPK_342620.1	1473.72
eS21	Y	159 aa	3-90	LdBPK_110780.1	132.65
eS24	Z	137 aa	3-129	LdBPK_363020.1	743.18
eS25	a	120 aa	35-106	LdBPK_251220.1	892.79
eS26	b	112 aa	2-104	LdBPK_280570.1	969.61
eS27	c	86 aa	2-85	LdBPK_363940.1	1695.62
eS28	d	87 aa	21-85	LdBPK_261610.1	2982.01
eS30	e	66 aa	2-66	LdBPK_300710.1	-
eS31	f	152 aa	83-147	LdBPK_360660.1	362.43
LACK1	g	312 aa	1-309	LdBPK_282970.1	4409.62

b.

Universal name	Chain ID	Length	Modeled range	TritrypDB ID	MS score (Mascot)
Alpha 26S	1	1782 b	3-1782	LdBPK_27rRNA4	-
Beta 26S	2	1526 b	2-1527	LinJ.27.rRNA1	-
Gamma 26S	3	213 b	2-213	LdBPK_27rRNA3	-
Delta 26S	4	183 b	1-183	LinJ.27.rRNA2	-
Epsilon 26S	5	133 b	1-132	LmjF.27.rRNA.43	-
Zeta 26S	6	76 b	3-73	-	-
5.8S	7	171 b	1-169	LdBPK_27rRNA5	-
5S	8	121 b	3-120	LdBPK_23rRNA1	-
P0	-	108 aa	-	LdBPK_150430.1	602.82
P1,P2	-	105 aa	-	LdBPK_303780.1	1981.39
uL1	-	78 aa	-	LdBPK_363950.1	1425.56
uL2	A	260 aa	2-257	LdBPK_351450.1	1100.8
uL3	B	419 aa	3-403	LdBPK_323320.1	461.9
uL4	C	373 aa	4-305	LdBPK_291160.1	4788.7
uL5	D	188 aa	8-175	LdBPK_220004.1	2077.3
uL6	E	190 aa	2-187	LdBPK_211290.1	2815.1
eL6	F	195 aa	20-195	LdBPK_330770.1	537.9
eL8	G	348 aa	111-341	LdBPK_070550.1	4773.5
uL11	-	164 aa	-	LdBPK_352230.1	2766.7
uL13	H	222 aa	5-222	LdBPK_340910.1	393.67
eL13	I	220 aa	2-207	LdBPK_292580.1	163.52
uL14	J	139 aa	10-139	LdBPK_353840.1	5275.24
eL14	K	233 aa	61-209	LdBPK_221370.1	974.92
uL15	L	145 aa	2-145	LdBPK_353810.1	723.71
eL15	M	204 aa	2-204	LdBPK_303710.1	2914.74
uL16	N	213 aa	1-203	LdBPK_040750.1	1565.46
uL18	O	305 aa	4-266	LdBPK_351870.1	1299.83
eL18	P	198 aa	4-198	LdBPK_364730.1	1538.97
eL19	Q	245 aa	2-189	LdBPK_060410.1	1214.43
eL20	R	179 aa	3-141	LdBPK_350600.1	1363.47
eL21	S	159 aa	2-158	LdBPK_343440.1	1711.16
uL22	T	166 aa	3-155	LdBPK_240040.1	2024.69
eL22	U	122 aa	2-122	LdBPK_363430.1	1319.34
uL23	V	145 aa	27-144	LdBPK_060600.1	2286.36
uL24	W	143 aa	2-124	LdBPK_242140.1	602.39
eL24	X	124 aa	2-65	LdBPK_361130.1	55.22
eL27	Y	134 aa	3-134	LdBPK_322850.1	1612.83
eL28	Z	147 aa	2-135	LdBPK_111110.1	758.34
uL29	a	127 aa	3-126	LdBPK_262350.1	269.04
eL29	b	70 aa	2-70	LdBPK_363550.1	292.42
uL30	c	252 aa	26-252	LdBPK_260160.1	2059.04
eL30	d	104 aa	8-100	LdBPK_350240.1	1647.18
eL31	e	183 aa	64-182	LdBPK_353340.1	1929.41
eL32	f	133 aa	2-131	LdBPK_212090.1	805.97
eL33	g	145 aa	20-144	LdBPK_342240.1	500.68
eL34	h	168 aa	3-127	LdBPK_363930.1	296.77
eL36	i	106 aa	3-100	LdBPK_210800.1	866.33
eL37	j	83 aa	2-81	LdBPK_332070.1	171.75
eL38	k	83 aa	2-76	LdBPK_262230.1	604.23
eL39	l	51 aa	2-51	LdBPK_161220.1	104.41
eL40	m	128 aa	77-127	LdBPK_311930.1	96.93
eL41	n	34 aa	1-34	-	-
eL43	o	92 aa	2-92	LdBPK_362020.1	688.30
eL44	p	106 aa	2-97	LdBPK_333380.1	301.46

Supplementary Table 3 | rRNA modifications

Modification		Position/chain	SU
m1acpΨ	1-methyl-3-(3-amino-3-carboxypropyl) pseudouridine	1543/**18S**	SSU
m5C	5-methylcytidine	2061/**18S**	
m7G	7-methylguanosine	1995/**18S**	
m4Cm	N4,2'-O-dimethylcytidine	2059/18S	
		2184/**18S**	
m62A	N6,N6-dimethyladenosine	2185/**18S**	
Am	2'-O-methyladenosine	28/**18S**	SSU
		98/**18S**	
		479/**18S**	
		668/**18S**	
		912/**18S**	
		2021/**18S**	
		69/**α**	LSU
		235/**α**	
		437/**α**	
		678/**α**	
		681/**α**	
		697/**α**	
		858/**α**	
		927/**α**	
		955/**α**	
		1541/**α**	
		382/**β**	
		502/**β**	
		527/**β**	
		570/**β**	
		572/**β**	
		591/**β**	
		604/**β**	
		628/**β**	
		665/**β**	
		1068/**β**	
		1186/**β**	
		1373/**β**	
		1385/**β**	
		43/**5.8S**	
		162/**5.8S**	
Gm	2'-O-methylguanosine	509/**18S**	SSU
		1464/**18S**	
		1478/**18S**	
		1550/**18S**	
		1623/**18S**	
		1647/18S	
		1829/**18S**	
		1865/**18S**	
		2151/**18S**	
		856/**α**	LSU
		959/**α**	
		1190/**α**	
		1526/**α**	
		1542/**α**	
		1628/**α**	
		71/**β**	
		534/**β**	
		641/**β**	
		655/**β**	
		1047/**β**	
		1079/**β**	
		1230/**β**	
		1232/**β**	
		1254/**β**	
		1361/**β**	
		74/**δ**	
		75/**5.8S**	

Modification		Position/chain	SU
Cm	2'-O-methylcytidine	18/**18S**	SSU
		38/**18S**	
		115/**18S**	
		1866/**18S**	
		2140/**18S**	
		695/**α**	LSU
		1529/**α**	
		359/**β**	
		443/**β**	
		583/**β**	
		1160/**β**	
		1249/**β**	
		1318/**β**	
		1398/**β**	
Um	2'-O-methyluridine	8/**18S**	SSU
		33/**18S**	
		661/**18S**	
		1621/**18S**	
		1777/**18S**	
		1833/**18S**	
		1979/**18S**	
		2048/**18S**	
		48/**α**	LSU
		845/**α**	
		847/**α**	
		1107/**α**	
		1253/**α**	
		1371/**α**	
		1661/**α**	
		73/**β**	
		560/**β**	
		667/**β**	
		1078/**β**	
		1153/**β**	
		1360/**β**	
		1420/**β**	
		10/γ	
		7/**5.8S**	
Ψ	pseudouridine	12, 33, 104, 455, 607, 609, 721, 1156,1192,1246, 1292,1371,1374, 1533,1559, 1566, 1657,1841, 1970, 2046, 2048, 2071 /**18S**	SSU
		672, 870, 940, 1011,1017,1055, 1084,1093,1171, 1181,1402,1530, 1535, 1666 /**α**	LSU
		437, 472, 500, 504, 506, 510, 512, 593, 595, 611, 662, 704, 1059,1061, 1144, 1214,1265, 1282, 1285,1304, 1319, 1355,1362, 1383, 1404, 1414 /**β**	
		69, 74 /**5.8S**	

2’-O-methylation and PSI positions were all validated by the presence of snRNA in the genome guiding the modifying enzymes to the relevant positions in the ribosome[4]

Supplementary Table 4 | 18S rRNA unique base modifications MS/MS confirmation

Modification	Position	MS inspection	(Candidate) position(s)	T1 fragment: sequence	T1 fragment: (candidate) position(s)	Error (ppm)	MW theo	MW obs
**m^{1}acp^{3}Ψ**	1543	m^{1}acp^3 Ψ	1543	AC(m1acp3Ψ)CAACAC(Gm)G	1541-1551	0.5	3679.585	3679.587
**m^{7}G**	1995	mG	1995	(mG)AAUG	1995-1999	0.6	1686.251	1686.252
**m^{4}Cm***	2059	Cm	755, 1345, **<u>2059</u>**, 2119	(Cm)C(mC)G	755-758, 1345-1348, **<u>2059-2062</u>**, 2119-2122	1.0	1306.213	1306.215
**m^{5}C**	2061	mC	757, 1347, **<u>2061</u>**, 2121	(Cm)C(mC)G	755-758, 1345-1348, **<u>2059-2062</u>**, 2119-2122	1.0	1306.213	1306.215
**m$^6_2$A**	2184	m$_2$A	2184	(m^{2}A)(m^{2}A)CCUG	2184-2189	0.0	1993.334	1993.334
**m$^6_2$A**	2185	m$_2$A	2185	(m^{2}A)(m^{2}A)CCUG	2184-2189	0.0	1993.334	1993.334

* The cryo-EM density indicates two methyl groups installed on the modified nucleotide, one at the 2'-O position of the sugar ring and an additional methyl at position 4 of the base (**Fig. 3C** in the main manuscript). Nevertheless, LC-MS results (further presented in **Supplementary Figures S7-S8**) indicated the mass of only one methyl group.

<u>Remark:</u> In addition to the reported base modifications, two base methylations were observed and modelled in the cryo-EM density map at σ=3.0. However, as their presence was not conferred by MS, we did not include those in the modification report. The modified residues are both belonging to the SSU 18S and include an m^{1}Ψ (1-methylpseudouridine) and an m^{5}C (5-methylcytidine) modification at positions 1539 and 1544, respectively.

Supplementary References

1 Borovinskaya, M. A. *et al.* Structural basis for aminoglycoside inhibition of bacterial ribosome recycling. *Nature structural & molecular biology* 14, 727-732, doi:10.1038/nsmb1271 (2007).

2 Wasserman, M. R. *et al.* Chemically related 4,5-linked aminoglycoside antibiotics drive subunit rotation in opposite directions. *Nature communications* 6, 7896, doi:10.1038/ncomms8896 (2015).

3 Matt, T., *et al.* Dissociation of antibacterial activity and aminoglycoside ototoxicity in the 4-monosubstituted 2-deoxystreptamine apramycin. *Proceedings of the National Academy of Sciences of the United States of America* 109, 10984-10989, doi:10.1073/pnas.1204073109 (2012).

4 Eliaz, D., et al. Genome-wide analysis of small nucleolar RNAs of Leishmania major reveals a rich repertoire of RNAs involved in modification and processing of rRNA. *RNA Biol.* 12(11), 1222-55, doi: 10.1080/15476286.2015.1038019 (2015).
